# Comparison of urban-rural inequality in quality antenatal care among women in Bangladesh and Pakistan: a multivariable decomposition analysis

**DOI:** 10.1186/s12978-026-02266-4

**Published:** 2026-01-20

**Authors:** Farjana Misu, Taslima Rahman, Dominic Gasbarro, Khurshid Alam

**Affiliations:** 1 Murdoch Business School, Murdoch University, Perth, WA 6150 Australia; 2 Department of Statistics, Jagannath University, Dhaka, 1100 Bangladesh; 3 Institute of Health Economics, University of Dhaka, Dhaka, 1000 Bangladesh

**Keywords:** Quality antenatal care, Urban-rural inequality, Blinder-Oaxaca multivariable decomposition, Bangladesh, Pakistan

## Abstract

**Background:**

Urban-rural inequality in accessing quality antenatal care (ANC) is a well-documented challenge in low- and middle-income countries like Bangladesh and Pakistan, hindering maternal healthcare utilization and progress towards the Sustainable Development Goals. This study explores the key factors contributing to this inequality in Bangladesh and Pakistan and highlights inter-country differences.

**Methods:**

We analyzed data from Demographic Health Surveys (2017–2018) of Bangladesh and Pakistan for women aged 15–49 who had at least one live birth in the three years preceding the survey. To identify the extent and sources of inequality, we decomposed urban-rural differences in quality ANC utilization into explained (attributable to variations in socioeconomic and demographic characteristics) and unexplained (reflecting differences in the effects of these characteristics) components using Blinder-Oaxaca type models adapted for nonlinear response variables.

**Results:**

Urban women were significantly more likely to receive quality ANC than rural women in both Bangladesh and Pakistan, with disparities of about 20%-25% points. Most of the inequality was explained by differences in socioeconomic and educational characteristics rather than behavioral factors. Wealth status was the dominant contributor, explaining nearly 58% of the inequality in Bangladesh and 46% in Pakistan, followed by women’s and husbands’ education, media exposure, and women’s autonomy. The pattern of predictors was broadly consistent across both countries, though education contributed more in Pakistan, while media exposure and husband’s education played a larger role in Bangladesh.

**Conclusion:**

Significant urban-rural inequality exists in Bangladesh and Pakistan, which is more pronounced in Pakistan. Among the common significant predictors for both countries, wealth disparity has the highest contribution percentage. In Pakistan, women’s education is the second largest contributor to inequality, whereas in Bangladesh, both media exposure and husband’s education played notable roles. Reducing urban-rural inequality in quality ANC requires targeted policies addressing wealth and educational disparities, along with interventions that promote media access and women’s autonomy to ensure equitable maternal healthcare utilization.

**Supplementary Information:**

The online version contains supplementary material available at 10.1186/s12978-026-02266-4.

## Background

Maternal morbidity and mortality, which are largely preventable, continue to be major health challenges worldwide [1]. In 2020, the global Maternal Mortality Ratio (MMR) was estimated at 223 per 100,000 live births, with low- and middle-income countries (LMICs) disproportionately bearing the burden [2, 3]. South Asia alone contributes 16% of global maternal deaths, highlighting the disparities in antenatal care (ANC) utilization and its direct impact on maternal health [3–5]. Access to quality ANC services, defined as receiving at least four ANC visits from a medically trained provider that include core clinical components such as blood pressure measurement, urine and blood sample collection, and counselling on pregnancy complications, is critical in preventing complications and improving maternal and neonatal outcomes [6]. This operational definition captures both the adequacy (frequency and provider competence) and comprehensiveness (range of services received) dimensions of ANC quality. However, significant inequalities in ANC utilization, particularly in LMICs like Bangladesh and Pakistan, remain a persistent issue [7–9].

While healthcare utilization has improved in many LMICs, the quality of care is often suboptimal [10]. Poor-quality ANC has been empirically linked to preventable maternal and neonatal deaths, low birth weight, stillbirths, and complications such as eclampsia and sepsis [11–13]. Studies have shown that women who receive inadequate ANC or care lacking essential components are at greater risk of maternal complications, intrapartum emergencies, and neonatal mortality [12, 14]. Conversely, quality ANC helps prevent hypertension, diabetes, pre-eclampsia, and infections, which, if undiagnosed, can lead to adverse maternal outcomes [15]. It also supports early detection of high-risk pregnancies and promotes institutional deliveries, thereby improving survival outcomes for both mothers and newborns [16, 17]. With the rise of noncommunicable diseases, high-quality ANC has become even more crucial for women with chronic illnesses [18].

There are marked disparities in ANC utilization within and between regions, particularly between urban and rural populations in South Asian countries [19]. Studies reveal pro-rich inequalities in maternal healthcare services (MHS), including skilled birth attendance (SBA) and institutional deliveries [10, 20–22]. Wealth, education, and location are key determinants influencing access to maternal healthcare in Bangladesh and Pakistan [23, 24]. In Bangladesh, factors such as religion, rural residency, and spousal education contribute significantly to inequity in ANC service use [9]. Similarly, in Pakistan, limited education, autonomy, high birth order, and rural residency hinder ANC utilization [25].

Globally, the coverage of ANC has improved, with 80% of women in LMICs receiving at least four ANC visits between 2016 and 2022 [26]. However, South Asia still lags, with only 55% of women receiving adequate ANC in 2022 [26]. Disparities are particularly evident in rural areas: in Bangladesh, 43% of rural women received at least four ANC visits compared to 59% in urban areas, and in Pakistan, rural women were 29% less likely to receive adequate ANC than their urban counterparts [27, 28]. These urban-rural inequalities reflect broader socio-economic disparities and healthcare access issues in both countries.

The urban-rural inequality in ANC utilization in Bangladesh and Pakistan is a pressing issue, given that over 60% of the population in both countries reside in rural areas [29, 30]. Both countries face similar public health challenges, including disparities in healthcare access, socio-economic inequalities in healthcare utilization, and limited healthcare infrastructure [31]. Comparing these two countries offers an opportunity to understand how similar health systems respond differently to structural inequities and to identify cross-country lessons applicable to other South Asian contexts with comparable demographic and institutional features.

Previous studies have largely examined ANC inequalities within single countries or focused on service coverage rather than service quality. Few have undertaken cross-country comparative analyses using quantitative decomposition techniques to isolate the determinants of ANC inequality in South Asia [32, 33]. This study identifies and compares the key contributors to urban-rural differences in quality ANC utilization in Bangladesh and Pakistan by applying the Blinder-Oaxaca multivariable decomposition approach (for a nonlinear response model) to Demographic and Health Survey (DHS) data from 2017 to 2018 for both countries. This decomposition approach is widely used to analyze group differences in healthcare utilization, to identify key factors contributing to these inequalities [34–39].

The findings of this study aim to inform the policymakers about the key modifiable factors driving urban-rural inequality in quality ANC services in Bangladesh and Pakistan. These insights are important for designing targeted interventions to reduce this gap and advance progress towards SDG 3 (ensuring healthy lives for all) and SDG 10 (reducing inequalities within and among countries) by 2030. Furthermore, comparing these two countries offers an opportunity to understand how similar health systems respond differently to structural inequities and to identify lessons that may inform analyses in other South Asian settings with comparable demographic and institutional characteristics.

## Methods

### Data source

The study used the DHS datasets from Bangladesh and Pakistan, collected during 2017–2018. Specifically, we utilized the Individual Recode (IR) dataset, which contains detailed information on women of reproductive age (15–49 years). These DHS (cross-sectional) surveys, essential for evidence-based policymaking, provide high-quality data on health, nutrition, and demographics [27, 28]. Both DHS surveys follow standardized protocols developed by the DHS Program to ensure comparability across countries and survey rounds [27, 28]. Each country’s DHS employs a stratified two-stage cluster sampling design to generate nationally representative samples; details of sampling, data collection, and weighting are available in the respective country reports [27, 28].

To ensure valid cross-country comparability, variable coding, value categories, and indicator definitions were reviewed and harmonized in accordance with DHS recode manuals. Any non-comparable variables were excluded, and consistency checks were conducted to verify alignment in value distributions and missing data patterns across datasets.

The National Research Ethics Committee of the Bangladesh Medical Research Council and the ICF Macro Institutional Review Board granted ethical approval for the Bangladesh Demographic and Health Survey (BDHS) 2017–2018. ICF Institutional Review Board, Pakistan Health Research Council, and National Bioethics Committee approved the Pakistan Demographic and Health Survey (PDHS) 2017–2018 protocol.

### Sample size

BDHS effectively interviewed 20,127 out of 20,376 women aged 15–49, yielding a 98.8% response rate [1]. With a response rate of 94.3% [2], the PDHS interviewed 12,364 out of 13,118 women aged 15–49 years [2]. In this study, we limited our sample to women who had given birth to at least one live birth in the three years preceding the survey. This recall period follows the standard DHS analytical convention and minimizes recall bias while maintaining a sufficiently large sample for reliable estimation. For women with more than one live birth during this period, information from the most recent birth was used to avoid duplication.

**Fig. 1:**
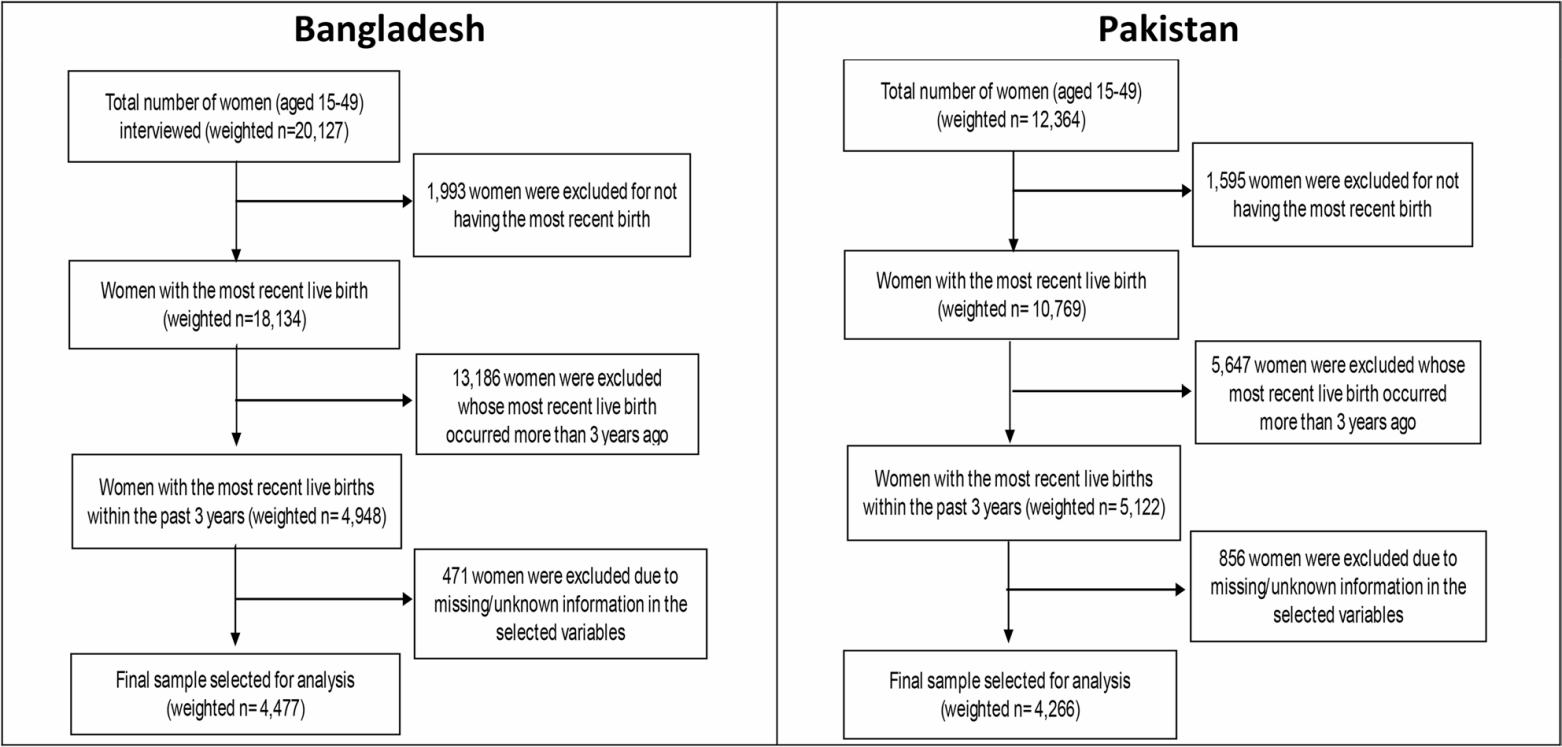
Sample selection for Bangladesh and Pakistan

Observations with missing values on the outcome or key independent variables were excluded through listwise deletion, as the proportion of missing data was minimal and randomly distributed. After applying these inclusion and exclusion criteria, the final analytical samples consisted of 4,477 women from Bangladesh and 4,266 women from Pakistan. Figure 1 illustrates the sample selection process for both countries.

### Outcome variable

The primary outcome of interest of the study is the utilization of quality ANC among women with at least one live birth in the last three years preceding the survey. The 2017-18 BDHS defined “quality ANC” as follows: a woman has four or more ANC visits, of which at least one is with a medically trained provider and receives all the basic components of ANC (weight and blood pressure measurements, urine and blood tests, and information on signs of possible complications) at least once. Previous studies have used similar constructs with slight variations. Anindya et al. [7] specified quality-adjusted ANC as ‘use at least four ANC services and receive key components of ANC: blood pressure taken, a urine sample taken, a blood sample taken, given iron tablets/ syrup’. Kim et al. [40] considered quality-adjusted ANC as women receiving at least four ANC visits and five basic services at any point during ANC: blood pressure measured, urine and blood samples collected, given, or bought iron tablets or syrup, and counselled on potential complications during pregnancy.

The 2017-18 PDHS did not explicitly define “quality ANC” but measured almost all the basic components of ANC except information on signs of possible complications during ANC visits. To ensure cross-country comparability, we harmonized the quality ANC measure by selecting only those indicators that were available and consistently defined in both surveys. Accordingly, quality ANC in this study was defined as having four or more ANC visits (with at least one by a medically trained provider) and receiving the following core components at least once: blood pressure measurement, and urine and blood sample collection.

Variables that were not uniformly available or defined across both datasets such as information on signs of complications, and iron supplementation were excluded to maintain methodological consistency. While this harmonization may narrow the conceptual scope of “quality ANC,” it ensures valid and reliable cross-country comparison between Bangladesh and Pakistan by focusing on universally measurable and clinically essential service components.

### Independent variables

The key independent variable in this study is women’s place of residence (urban or rural), which serves as the primary stratifying factor for assessing inequality in the utilization of quality ANC services. Differences in women’s socio-economic and demographic characteristics between urban and rural areas are expected to contribute to variations in quality ANC service utilization.

**Fig. 2:**
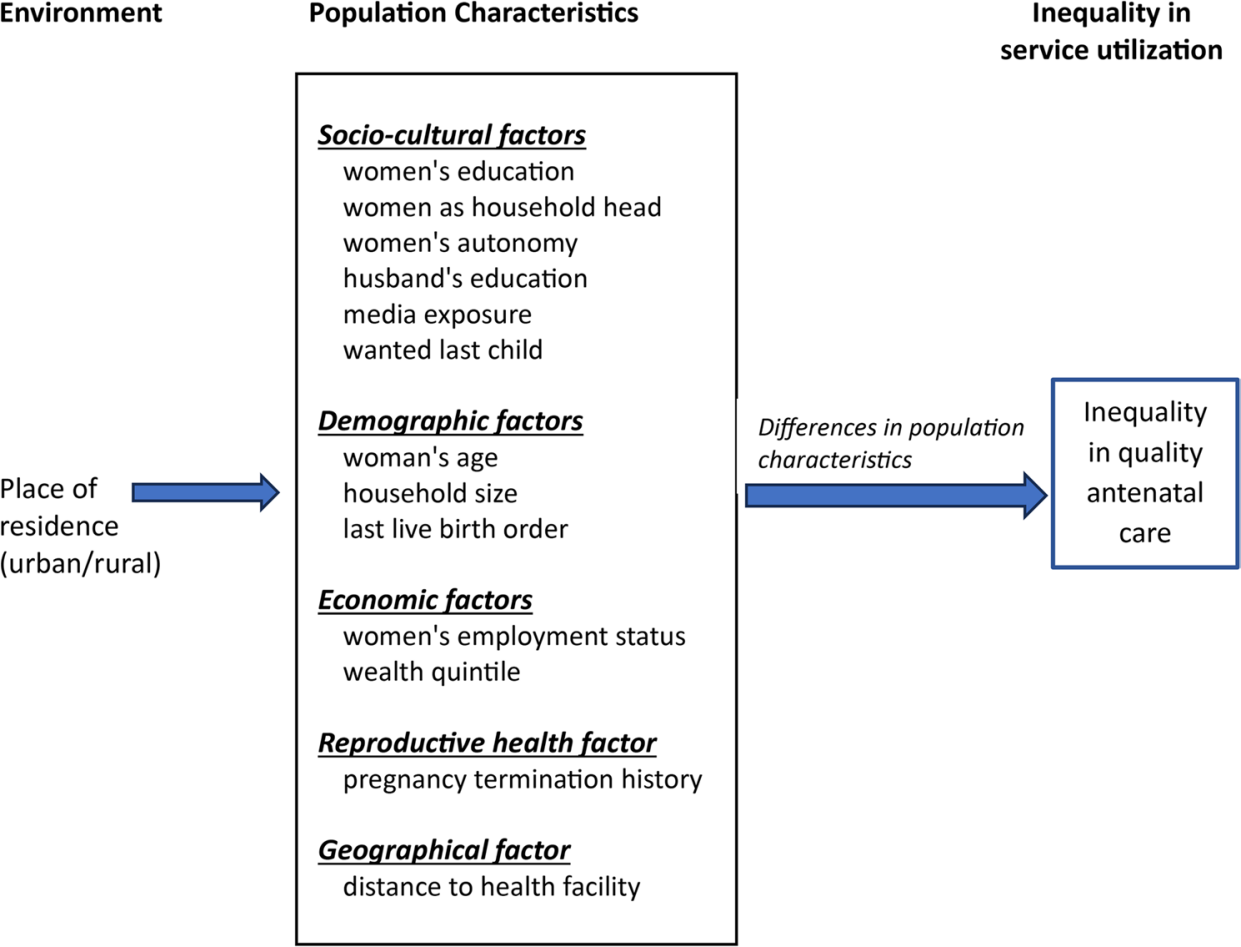
Conceptual framework of inequality in quality ANC service utilization

Guided by the conceptual framework (see Fig. 2), additional explanatory variables were included to account for these contextual differences. Population characteristics were grouped into five components: socio-cultural factors (women’s education, women as household head, women’s autonomy, husband’s education, media exposure, and wanted last child), demographic factors (woman’s age, household size, last live birth order), economic factors (women’s employment status, wealth quintile), reproductive health factor (pregnancy termination history), and geographic factor (distance to health facility).

Since, the utilization of antenatal care and maternal healthcare services does vary significantly between younger, middle-aged, and older women, we categorized the age variable into three groups, following the age categorization used in BDHS and PDHS reports [41–43]. The outcome and independent variables are classified, levelled, and coded in Supplementary Table 1.

### Statistical and decomposition analysis

We conducted the empirical analyses in three stages. Initially, we performed descriptive analyses of women’s background characteristics by place of residence. We implemented the Pearson chi-square ($\:{\chi\:}^{2}$) test to identify any significant difference between the proportion of women accessing quality ANC utilization in urban and rural areas. The Pearson chi-square ($\:{\chi\:}^{2}$) test was also used to examine whether women’s place of residence (urban/rural) was statistically different for categorical independent variables. Variance Inflation Factors (VIF) for all covariates were examined to address potential collinearity, with the results (VIF < 10) confirming that multicollinearity is not a concern among the independent variables. Supplementary Table 2 provides the VIF values for the variables included in the decomposition models.

In the final stage, a decomposition framework was used to quantify the contribution of observed characteristics (explained component) and differences in the effects of those characteristics (unexplained component) to the overall urban-rural gap in quality ANC utilization. The extended Blinder-Oaxaca decomposition for nonlinear outcomes, developed by Powers et al. [44], served as the primary analytical approach. Its main applications include non-linear decomposition and convenient methods to handle path dependency [45], and solving the identification issue in choosing a reference category when dummy variables are included. In this detailed decomposition analysis, we use the *mvdcmp* command along with normal and logit codes. In the decomposition analysis, we incorporated all the independent variables, which exhibited statistically significant urban-rural differences at a 5% level in either country. To assess the robustness of the estimates in the detailed decomposition, we repeated the analysis by excluding covariates that were not statistically significant in the primary analysis.

To ensure robustness and methodological triangulation, we applied four decomposition techniques, Oaxaca, Blinder, Reimers, and Cotton to decompose urban-rural differences in the utilization of quality ANC. The Oaxaca and Blinder methods differ in reference group selection (high versus low performing group), while the Reimers and Cotton approaches apply alternative weighting schemes (equal or sample proportion-based weights). Because decomposition outcomes can vary depending on the choice of weights, employing multiple methods enabled robustness testing and methodological triangulation, ensuring that observed results were not driven by a specific weighting assumption. Consistent findings across all approaches strengthened the credibility of the results.

All analyses were conducted separately for Bangladesh and Pakistan using STATA 17.0, with appropriate sampling weights applied to account for the DHS complex survey design. The detailed mathematical derivations and equations for the decomposition models are presented in Supplementary File 1.

## Results

### Distribution of background characteristics and urban-rural in quality ANC

**Fig. 3:**
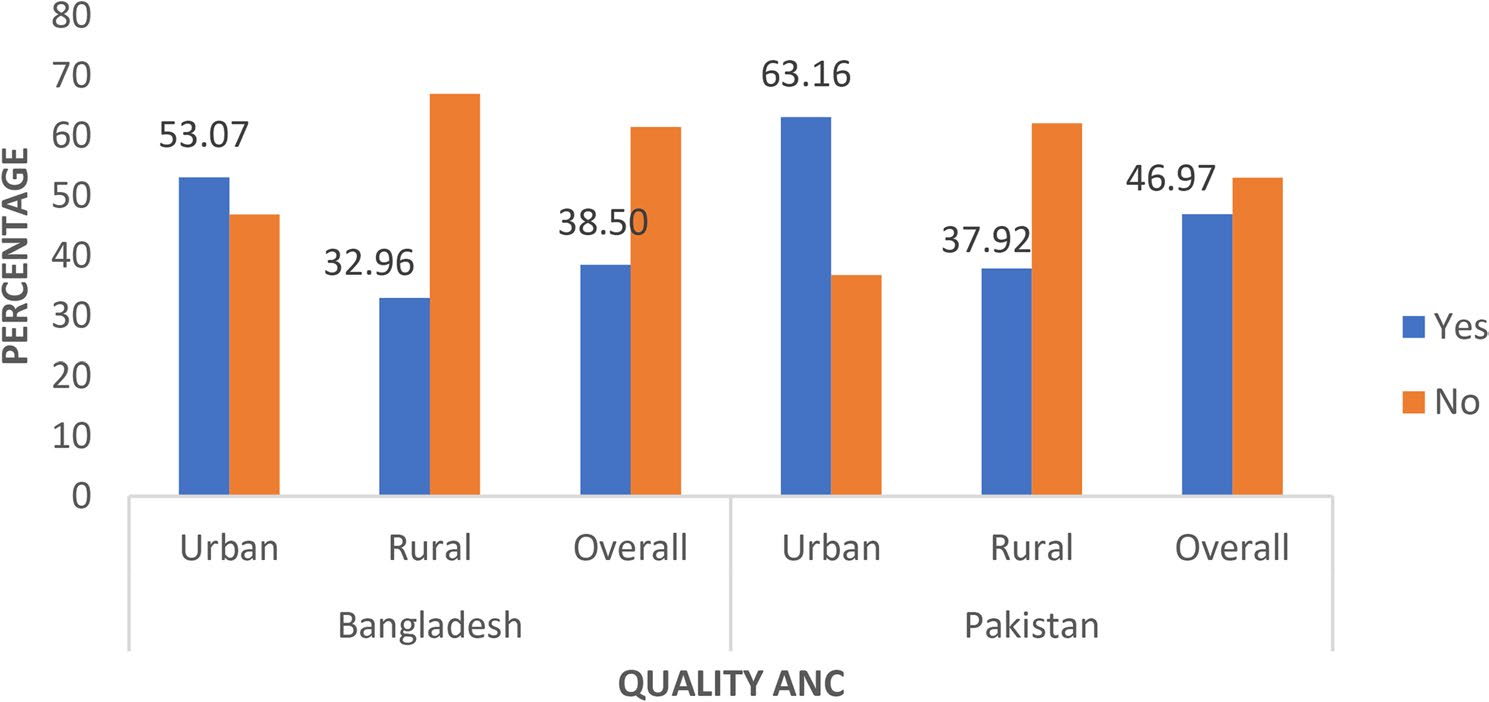
Proportion of women utilizing quality ANC by place of residence in Bangladesh and Pakistan, DHS 2017-18

Figure 3 illustrates the overall proportion of women utilizing quality ANC by place of residence (urban-rural) in Bangladesh and Pakistan. Overall, 38.50% of women in Bangladesh received quality ANC. About 53.07% of urban women in Bangladesh utilize quality ANC, compared to 32.96% of rural women. The utilization rate of quality ANC in Pakistan was recorded at 46.97%. In Pakistan, approximately 63.16% of urban women utilized quality ANC, while rural women lagged behind by 37.92%. The difference in quality ANC uses by place of residence (urban-rural) within each county is statistically significant ($\:{\chi\:}_{\left(1\right)}^{2}$, $p-value\le 0.01$).

Table 1 shows women’s background characteristics by place of residence (urban/ rural) in Bangladesh and Pakistan. We did a Pearson chi-square test to identify significant differences among independent variables by place of residence (Table 1). In Bangladesh, the completion of secondary or higher education for rural women and their husbands was around 21% and 22%, respectively, while in urban, it was 34% and 36%, respectively. Around half of the rural women had media exposure (48%) and decision-making autonomy (51%), while in urban areas, two-thirds (72%) of women had media exposure, and around 60% had decision-making autonomy. About 76% of rural women and 77% of urban women were in the 20–34 years age category. Only 10% of women in rural areas belonged to the richest wealth quintile, compared to 43% in urban areas.

In Pakistan (Table 1), the completion of secondary or higher education for rural women and their husbands was around 15% and 33%, respectively, while in urban, it was 40% and 56%, respectively. Around 38% of rural women had media exposure, and 26% had decision-making autonomy, while among urban women, 65% had media exposure, and 31% had decision-making autonomy. Most women (77% in rural and 80% in urban) were in the 20–34 years age category. Only 9% of women in rural areas were in the richest wealth quintile, compared to about 38% of women in urban areas. Except for the wanted last child variable, all the independent variables in Table 1 exhibited a significant urban-rural difference ($\:{\chi\:}^{2}\:$test, $\:p-value\le\:0.05$) for at least one of the countries.

**Table 1:** Background characteristics of women by place of residence in Bangladesh and Pakistan, DHS 2017-18

Characteristics	Bangladesh		Pakistan	
Residence	Rural (%)	Urban (%)	Rural (%)	Urban (%)
National	64.55	35.45	49.84	50.16
**Socio-cultural factors**				
** Women’s education**	$\:{\chi\:}_{\left(4\right)}^{2}\:$= 93.2**		$\:{\chi\:}_{\left(4\right)}^{2}\:$= 415.6**	
No formal education	4.95	4.73	59.60	34.44
Primary education not completed	16.82	14.56	6.40	3.41
Primary education completed	11.18	8.44	9.83	9.44
Junior school completed	46.33	38.56	9.36	13.27
Secondary or higher	20.73	33.71	14.82	39.44
** Women as household head**	$\:{\chi\:}_{\left(1\right)}^{2}\:$= 19.9**		$\:{\chi\:}_{\left(1\right)}^{2}\:$= 23.4**	
Yes	13.53	9.01	11.85	7.48
** Women’s autonomy**	$\:{\chi\:}_{\left(1\right)}^{2}\:$= 28.2**		$\:{\chi\:}_{\left(1\right)}^{2}\:$= 13.0**	
Yes	51.28	59.55	25.82	30.79
** Husband’s education**	$\:{\chi\:}_{\left(4\right)}^{2}\:$= 114.2**		$\:{\chi\:}_{\left(4\right)}^{2}\:$= 246.1**	
No formal education	14.01	9.26	32.55	17.24
Primary education not completed	19.65	15.19	5.88	4.21
Primary education completed	14.91	12.85	10.87	8.22
Junior school completed	29.76	26.78	18.02	14.86
Secondary or higher	21.66	35.92	32.69	55.47
** Media exposure**	$\:{\chi\:}_{\left(1\right)}^{2}\:$= 237.2**		$\:{\chi\:}_{\left(1\right)}^{2}\:$= 315.6**	
Yes	48.44	72.27	38.19	65.37
** Wanted last child**	$\:{\chi\:}_{\left(1\right)}^{2}\:$= 1.4		$\:{\chi\:}_{\left(1\right)}^{2}\:$= 1.9	
Yes	79.86	78.39	89.46	88.13
**Demographic factors**				
** Woman’s age (years)**	$\:{\chi\:}_{\left(2\right)}^{2}\:$= 10.2**		$\:{\chi\:}_{\left(2\right)}^{2}\:$= 3.8	
15–19	18.75	15.75	5.83	4.95
20–34	76.02	77.38	77.42	79.81
35–49	5.81	6.87	16.75	15.23
** Household size**	$\:{\chi\:}_{\left(1\right)}^{2}\:$= 17.1**		$\:{\chi\:}_{\left(1\right)}^{2}\:$= 0.1	
1–5 members	48.30	54.76	20.37	20.00
** Last live birth order**	$\:{\chi\:}_{\left(3\right)}^{2}\:$= 13.3**		$\:{\chi\:}_{\left(3\right)}^{2}\:$= 20.9**	
First	38.72	41.15	22.06	24.02
Second	32.25	34.59	21.26	24.58
Third	17.20	15.31	17.31	18.64
Fourth or higher	11.83	8.95	39.37	32.76
**Economic factors**				
** Employment status**	$\:{\chi\:}_{\left(1\right)}^{2}\:$= 66.4**		$\:{\chi\:}_{\left(1\right)}^{2}\:$= 10.1**	
Currently employed	40.93	28.67	12.79	9.72
** Wealth quintile**	$\:{\chi\:}_{\left(4\right)}^{2}\:$= 863.4**		$\:{\chi\:}_{\left(4\right)}^{2}\:$= 1100.0**	
Poorest	24.64	9.45	30.39	4.77
Poorer	26.12	8.00	25.73	10.42
Middle	21.00	13.30	22.15	18.46
Richer	17.85	25.90	12.75	28.74
Richest	10.38	43.35	8.98	37.62
**Reproductive health factor**				
** Pregnancy termination history**	$\:{\chi\:}_{\left(1\right)}^{2}\:$= 6.5**		$\:{\chi\:}_{\left(1\right)}^{2}\:$= 0.03	
Yes	16.02	19.03	28.17	27.94
**Geographical factor**				
** Distance to health facility**	$\:{\chi\:}_{\left(1\right)}^{2}\:$= 62.6**		$\:{\chi\:}_{\left(1\right)}^{2}\:$= 209.0**	
Big problem	43.84	31.76	55.55	33.55

** Significance at 1% level, * Significance at 5% level and χ^2^ = Pearson’s chi-square,Total women included in the analysis: Bangladesh = 4,477; Pakistan = 4,266

### Aggregate decomposition

Table 2 presents the twofold decomposition estimates of the urban-rural inequality in the utilization of quality ANC using four different methods for Bangladesh and Pakistan. In Bangladesh, the average total difference in predicted quality ANC rates by place of residence was 0.2012 (95% CI: 0.1707–0.2316) statistically significant at 1% level. This finding indicates that the utilization of quality ANC by urban women exceeded that of rural by 20.12% on average, regardless of the type of decomposition used. However, in Pakistan, the average total difference stood at 0.2524 (95% CI: 0.2173–0.2875), with the urban rate surpassing the rural by 25.24%, consistent across all decomposition methods employed. The analysis also reveals variations in the proportion of this disparity attributable to explained (due to differences in characteristics) and unexplained (due to differences in coefficients) components, depending on the weighting scheme used. However, substantial portions of the urban-rural gaps can be attributed to different characteristics across all methods. In both countries, difference due to characteristics account for most of the differences. The positive signs of these contributions indicate that aligning the covariate distributions of rural women with those of urban women could potentially eliminate the observed disparities in the quality ANC utilization.

According to Table 2, in Bangladesh, the explained component is approximately 87% ($\:p-value\le\:0.01$) when using the Oaxaca decomposition, compared to 50% ($\:p-value\le\:0.01$) when using the Blinder decomposition, suggesting that the favoritism towards urban women contributes more to the inequality in utilization of quality ANC than discrimination against rural women. In Pakistan, the Oaxaca decomposition yields 89% ($\:p-value\le\:0.01$), and the Blinder decomposition yields 85% ($\:p-value\le\:0.01$) for the explained component, indicating a similar trend. These results are confirmed by the Cotton and Reimers decompositions, which show how both favoritism toward urban women and discrimination against rural women contribute to inequality. Specifically, for Bangladesh, using the Reimers decomposition, about 24% ($\:p-value\le\:0.01$) of the inequality is explained by an advantage to urban women, while only 6% is explained by a disadvantage to rural women. Similarly, when using the Cotton decomposition, 17% ($\:p-value\le\:0.01$) of the inequality is explained by an advantage to urban women, compared to 8% explained by a disadvantage to rural women in Bangladesh. For Pakistan, according to Cotton decomposition, around 7% and 6% of the inequality is explained by advantages to urban and disadvantages to rural women, respectively. In Table 2, the weights of the Cotton decomposition were based on the sample proportions of women in rural areas (reference group), approximately 0.65 for Bangladesh and 0.50 for Pakistan.

**Table 2:** Twofold decomposition estimates of quality ANC by residence in Bangladesh and Pakistan, DHS 2017-18

Decomposition	Bangladesh			Pakistan		
	Coefficient	[95% CI]	Percent	Coefficient	[95% CI]	Percent
Oaxaca Decomposition weight = 1						
Explained	0.1749**	[0.1432, 0.2025]	86.95	0.2233**	[0.1719, 0.2387]	88.48
Unexplained	0.0263	[-0.01835, 0.0637]	13.05	0.0291	[-0.0220, 0.0747]	11.52
Total	0.2012**	[0.1620, 0.2290]	100.00	0.2524**	[0.1962, 0.2671]	100.00
**Blinder Decomposition weight = 0**						
Explained	0.1005**	[0.0814, 0.1245]	49.94	0.2136**	[0.1745, 0.2265]	84.63
Unexplained	0.1007**	[0.0535, 0.1316]	50.06	0.0388	[0.0092, 0.0715]	15.37
Total	0.2012**	[0.1620, 0.2290]	100.00	0.2524**	[0.1962, 0.2671]	100.00
**Reimers Decomposition: weight = 0.5**						
Explained	0.1393**	[0.1182, 0.1609]	69.27	0.2201**	[0.1810, 0.2275]	87.20
Advantage	0.0490**	[0.0261, 0.0638]	24.34	0.0182	[-0.0048, 0.0343]	7.21
Disadvantage	0.0129	[-0.0107, 0.0327]	6.39	0.0141	[-0.0117, 0.0371]	5.59
Total	0.2012**	[0.1577, 0.2333]	100.00	0.2524**	[0.1968, 0.2665]	100.00
**Cotton Decomposition**:	**weight = 0.65**			**weight = 0.50**		
Explained	0.1501**	[0.1301, 0.1693]	74.62	0.2201**	[0.1813, 0.2272]	87.20
Advantage	0.0343**	[0.0196, 0.0434]	17.06	0.0181	[-0.0038, 0.0331]	7.19
Disadvantage	0.0168	[-0.0095, 0.0383]	8.32	0.0142	[-0.0106, 0.0360]	5.61
Total	0.2012**	[0.1645, 0.2266]	100.00	0.2524**	[0.1960, 0.2674]	100.00

** Significance at 1% level, CI = Confidence Interval

### Detailed decomposition

Table 3 details the contributions of individual characteristics of women to the urban-rural inequality in the utilization of quality ANC in Bangladesh and Pakistan. In Bangladesh, differences in the distribution of characteristics (explained component) accounted for about 82.28% ($\:p-value\le\:0.01$) of the inequality in quality ANC utilization. In Pakistan, this figure was even higher at 92.85% ($\:p-value\le\:0.01$). For the unexplained component (differences due to the coefficient), we did not find any significant impact on urban-rural inequality in either country.

In Bangladesh, the detailed decomposition results (Table 3) demonstrated that wealth status was the predominant factor, explaining 57.94% of the disparity in quality ANC utilization. Specifically, the richest wealth quintile alone contributed 38.05% ($\:p-value\le\:0.01$) to the overall disparity, while the poorest quintile contributed 10.96% ($\:p-value\le\:0.01$). Other significant factors included women’s media exposure (8.92%, $\:p-value\le\:0.05$), husband’s secondary or higher education (7.55%, $\:p-value\le\:0.01$), women’s secondary and higher education (3.74%, $\:p-value\le\:0.05$), and women’s autonomy (2.34%, $\:p-value\le\:0.05$). The positive signs of these contributions indicate that aligning these covariate distributions of rural women with those of urban women in Bangladesh could eliminate the observed disparities in quality ANC utilization. Conversely, urban-rural difference in the proportion of women who had completed junior school contributed negatively to the overall disparity (-3.63%, $\:p-value\le\:0.01$). This suggests that if the distribution of this education level among rural women were aligned with that of urban women in Bangladesh, it could widen the urban-rural disparity in quality ANC utilization.

The detailed decomposition results (Table 3) for Pakistan also highlighted wealth status as a major contributor to inequality, with 45.93% attributed to this factor. The richest and the poorest wealth quintiles contributed 18.23% ($\:p-value\le\:0.01$)and 18.75% ($\:p-value\le\:0.01$), respectively. Aligning the rural distribution of other significant covariates such as women’s secondary and higher education (14.67%, $\:p-value\le\:0.01$), women’s media exposure (9.16%, $\:p-value\le\:0.01$), husbands’ secondary and higher education (6.69%, $\:p-value\le\:0.01$), women’s autonomy (2.16%, $\:p-value\le\:0.05$), and first-order birth (1.41%, $\:p-value\le\:0.01$), to those of urban women, could help to narrow the urban-rural inequality in the utilization of quality ANC in Pakistan.

**Table 3:** Detailed decomposition of quality ANC by place of residence for Bangladesh and Pakistan, DHS 2017-18

Decomposition	Bangladesh			Pakistan		
	Coefficient	[95% CI]	Percent	Coefficient	[95% CI]	Percent
Total difference	0.2012**	[0.1707, 0.2316]	100	0.2524**	[0.2173, 0.2875]	100
Explained	0.1655**	[0.1377, 0.1934]	82.28	0.2344**	[0.1947, 0.2740]	92.85
Unexplained	0.0357	[-0.0026, 0.0738]	17.72	0.0180	[-0.0333, 0.0693]	7.15
**Explained component = difference in characteristics (E)**						
Woman’s age (years)			0.53			-0.22
15–19	0.0008	[-0.0011, 0.0027]	0.40	0.0002	[-0.0008, 0.0012]	0.08
20–34	-0.0001	[-0.0010, 0.0008]	-0.04	-0.0002	[-0.0020, 0.0016]	-0.08
35–49	0.0003	[-0.0006, 0.0012]	0.17	-0.0006	[-0.0021, 0.0010]	-0.22
Women’s education			-0.23			22.84
No formal education	-0.0005*	[-0.0009, -0.0000]	-0.23	0.0169	[0.0004, 0.0335]	6.71
Primary education not completed	0.0003	[-0.0002, 0.0007]	0.14	0.0025*	[-0.0017, 0.0067]	0.98
Primary education completed	-0.0002	[-0.0024, 0.0021]	-0.09	0.0002	[-0.0004, 0.0008]	0.08
Junior school completed	-0.0073**	[-0.0115, -0.0031]	-3.63	0.0010	[-0.0026, 0.0046]	0.40
Secondary or higher	0.0075*	[-0.0002, 0.0153]	3.74	0.0370**	[0.0207, 0.0534]	14.67
Currently employed women	0.0014	[-0.0064, 0.0091]	0.68	0.0014	[-0.0028, 0.0056]	0.56
Women as household head	-0.0011	[-0.0055, 0.0033]	-0.56	-0.0008	[-0.0041, 0.0025]	-0.31
Women have media exposure	0.0179*	[0.0024, 0.0335]	8.92	0.0231**	[0.0048, 0.0415]	9.16
Women have autonomy	0.0047*	[0.0003, 0.0091]	2.34	0.0055*	[0.0006, 0.0103]	2.16
1–5 members in household	-0.0028	[-0.0070, 0.0013]	-1.39	0.0006	[-0.0000, 0.0012]	0.23
Wealth quintile			57.94			45.93
Poorest	0.0221**	[0.0090, 0.0351]	10.96	0.0473**	[0.0116, 0.0830]	18.75
Poorer	0.0112	[-0.0042, 0.0265]	5.56	0.0034	[-0.0111, 0.0178]	1.33
Middle	0.0041	[-0.0029, 0.0111]	2.05	0.0018	[-0.0047, 0.0082]	0.70
Richer	0.0027	[-0.0016, 0.0069]	1.32	0.0175**	[0.0073, 0.0276]	6.92
Richest	0.0765**	[0.0563, 0.0968]	38.05	0.0460**	[0.0237, 0.0683]	18.23
Distance is a big problem	0.0034	[-0.0045, 0.0113]	1.70	0.0051	[-0.0086, 0.0187]	2.02
Husband’s education			9.5			7.44
No formal education	0.0001	[-0.0034, 0.0035]	0.03	-0.0007	[-0.0118, 0.0104]	-0.27
Primary education not completed	0.0032*	[0.0005- 0.0060]	1.60	0.0003	[-0.0008, 0.0014]	0.11
Primary education completed	0.0005	[-0.0004, 0.0014]	0.25	0.0029*	[0.0004, 0.0053]	1.14
Junior school completed	0.0002	[-0.0014, 0.0017]	0.07	-0.0006	[-0.0017, 0.0006]	-0.23
Secondary or higher	0.0152**	[0.0070, 0.0234]	7.55	0.0169**	[0.0056, 0.0281]	6.69
Last live birth order			2.69			3.04
First	0.0015*	[0.0002, 0.0028]	0.75	0.0036**	[0.0011, 0.0061]	1.41
Second	-0.0005	[-0.0019, 0.0010]	-0.23	0.0010	[-0.0006, 0.0026]	0.39
Third	-0.0011	[-0.0023, 0.0001]	-0.55	-0.0001*	[-0.0002, -0.0000]	-0.04
Fourth or higher	0.0034*	[0.0005, 0.0063]	1.69	0.0032	[-0.0010, 0.0074]	1.27
Has Pregnancy termination history	0.0021	[-0.0005, 0.0047]	1.03	0.0000	[-0.0005, 0.0005]	0.01
**Unexplained component = difference in coefficients (C)**						
Woman’s age (years)						
15–19	-0.0040	[-0.0219, 0.0140]	-1.97	-0.0004	[-0.0102, 0.0095]	-0.14
20–34	0.0082	[-0.0435, 0.0599]	4.08	0.0033	[-0.0828, 0.0893]	1.29
35–49	0.0005	[-0.0059, 0.0069]	0.25	0.0005	[-0.0226, 0.0236]	0.19
Women’s education						
No formal education	0.0002	[-0.0081, 0.0084]	0.08	0.0077	[-0.0582, 0.0736]	3.06
Primary education not completed	0.0053	[-0.0106, 0.0212]	2.64	0.0006	[-0.0159, 0.0171]	0.23
Primary education completed	0.0005	[-0.0116, 0.0125]	0.23	-0.0032	[-0.0235, 0.0171]	-1.28
Junior school completed	0.0154	[-0.0205, 0.0513]	7.66	-0.0106	[-0.0478, 0.0266]	-4.20
Secondary or higher	-0.0155	[-0.0358, 0.0049]	-7.70	0.0174	[-0.0422, 0.0770]	6.89
Currently employed women	-0.0121	[-0.0454, 0.0211]	-6.03	-0.0069	[-0.0446, 0.0307]	-2.74
Women as household head	-0.0042	[-0.0208, 0.0124]	-2.08	0.0074	[-0.0253, 0.0400]	2.93
Women have media exposure	0.0102	[-0.0355, 0.0558]	5.06	0.0655	[-0.1555, 0.2864]	25.95
Women have autonomy	0.0471	[0.0009, 0.0933]	23.41	0.0105	[-0.0430, 0.0641]	4.17
1–5 members in household	-0.0258	[-0.0656, 0.0141]	-12.82	0.0331	[-0.0791, 0.1453]	13.11
Wealth quintile						
Poorest	-0.0184	[-0.0473, 0.0104]	-9.17	-0.0204	[-0.1512, 0.1104]	-8.08
Poorer	0.0008	[-0.0237, 0.0254]	0.42	0.0367	[-0.0952, 0.1687]	14.56
Middle	-0.0080	[-0.0261, 0.0100]	-3.99	0.0007	[-0.0342, 0.0357]	0.29
Richer	-0.0015	[-0.0147, 0.0116]	-0.75	0.0088	[-0.0337, 0.0513]	3.50
Richest	0.0133	[0.0020, 0.0246]	6.60	-0.0129	[-0.0510, 0.0253]	-5.10
Distance is a big problem	0.0039	[-0.0303, 0.0382]	1.95	0.0077	[-0.0608, 0.0763]	3.07
Husband’s education						
No formal education	0.0023	[-0.0120, 0.0167]	1.16	0.0311	[-0.0805, 0.1428]	12.33
Primary education not completed	-0.0057	[-0.023, 0.0116]	-2.83	-0.0031	[-0.0183, 0.0120]	-1.24
Primary education completed	0.0015	[-0.0112, 0.0142]	0.77	-0.0197	[-0.0880, 0.0485]	-7.82
Junior school completed	-0.0046	[-0.0249, 0.0157]	-2.27	0.0050	[-0.0243, 0.0342]	1.96
Secondary or higher	0.0036	[-0.0151, 0.0223]	1.78	0.0285	[-0.0690, 0.1259]	11.28
Last live birth order						
First	0.0132	[-0.0172, 0.0436]	6.57	0.0139	[-0.0384, 0.0661]	5.50
Second	-0.0137	[-0.0354, 0.0079]	-6.83	0.0067	[-0.0249, 0.0383]	2.66
Third	0.0103	[-0.0043, 0.0248]	5.11	-0.0204	[-0.0898, 0.0490]	-8.09
Fourth or higher	-0.0062	[-0.0208, 0.0084]	-3.08	0.0101	[-0.0443, 0.0645]	4.01
Has pregnancy termination history	-0.0016	[-0.0162, 0.0131]	-0.77	-0.0352	[-0.1557, 0.0852]	-13.96
Constant	0.0206	[-0.0904, 0.1316]	10.24	-0.1443	[-0.7072, 0.4187]	-57.16

Detailed decomposition through Blinder-Oaxaca multivariable decomposition devised by Powers et al.** Significance at 1% level, * Significance at 5% level and CI = Confidence Interval

In our robustness check, excluding non-statistically significant covariates reveals consistent results with the same set of contributing factors showing only minor variations in magnitude (Supplementary Table 3).

## Discussion

In this study, we investigated and determined the underlying factors behind the urban-rural inequality in the utilization of quality ANC services based on the DHS data of Bangladesh and Pakistan of the same year (2017–2018), using a Blinder-Oaxaca and related decomposition analysis. To our knowledge, this is the first study to explain and compare the observed inequality in the utilization of quality ANC services between urban and rural women in Bangladesh and Pakistan. Our findings offer insights that could shape policy decisions aimed at diminishing maternal health inequalities and improving population health outcomes in these countries.

We detected significant urban-rural inequality in the study countries (Fig. 3); in Pakistan, the urban-rural quality ANC inequality was marginally higher than in Bangladesh. This inequality may be attributed to the poor wealth status of rural women, the illiteracy of husbands, and less exposure to media in both countries but dominantly in Pakistan. Our descriptive statistics confirmed that in Pakistan, the majority of rural women belonged to the poorest wealth group, their husbands lacked formal education, and they had less media exposure, whereas, in urban settings, these proportions were considerably lower. Rich women in urban areas have easy access to information about maternal health through diverse media, enabling them to comprehend the importance of ANC services [37].

Our findings demonstrate that more than half of the observed inequality in the utilization of quality ANC among urban and rural women in Bangladesh and Pakistan can be attributed to differences in the distribution of quality ANC covariates between these groups, regardless of the decomposition types used (Table 2). This means that the contribution of composition (endowments) changes is more critical than behavior (coefficient) changes in reducing the inequality between urban and rural women’s use of quality ANC. This inequality could be because urban women are more likely to utilize ANC services than rural counterparts since they are better endowed with the factors that encourage quality ANC (rich wealth, both women’s and husbands’ higher education, access to media, and higher autonomy). Our descriptive results (Table 1) reinforced this explanation, which revealed that the percentage of wealth, higher education, media access and autonomy was higher among urban women than rural women. Positive coefficients (Table 3) imply that the inequality in quality ANC would narrow if rural women’s endowment were equivalent to that of their urban counterparts. This finding is further supported by results in five West-African countries, namely Burkina Faso, Niger, Nigeria, Ghana, and Senegal [46], in addition to Ethiopia [35] and Nepal [36]. Urban-rural inequality due to the differences in characteristics (covariate effect) is substantial in both countries, exceeding 80% in Bangladesh and 90% in Pakistan (Table 3). This suggests that a significant portion of urban-rural inequality could be minimized if appropriate policies were implemented to equalize differences in characteristics between these two countries, especially in Pakistan.

In Bangladesh and Pakistan, the inequality in the utilization of quality ANC can be primarily attributed to the wealth status difference, accounting for approximately half of the total inequality between urban and rural women. For Bangladesh, the wealthiest quintile contributes the highest among all covariate categories. This result suggests that reducing this wealth-based inequality by elevating the proportion of rural women to the urban level could mitigate the maximum inequality in the utilization of quality ANC services. For Pakistan, if the percentage of the richest and poorest group of women in rural were equalized to urban women, then it could reduce most of the inequality in quality ANC. This finding is also supported by an earlier study in Bangladesh and Pakistan [9], which reported that inequality in the utilization of ANC services was more concentrated among women with low economic status. Women of the richest wealth group can afford healthcare expenses, while the poorest women can barely afford out-of-pocket payments for any health crises [47]. Therefore, low economic condition impedes women’s access to critical maternal healthcare services and reinforces the rich-poor disparity in healthcare utilization.

Media exposure contributes to urban-rural inequality, both in Bangladesh and Pakistan. Enhancing media exposure for rural women to match that of their urban counterparts could reduce inequality in the utilization of quality ANC. This finding is also supported by results in Ethiopia [48], which implies that increasing media access for women in rural areas would effectively reduce the geographic inequalities in utilizing ANC. Frequent and targeted media campaigns could influence rural women’s ANC follow-ups by providing essential information about pregnancy risks, thereby eliminating service utilization inequality.

In Bangladesh and Pakistan, secondary or higher education of women and their husbands contributes to urban-rural inequality. These results imply that if the women and their husband’s education in rural areas were enhanced as women of urban areas, then the inequality in the utilization of quality ANC services could be minimized. Supporting evidence from Ghana indicates that improving education for women and their partners to at least a secondary level can significantly reduce disparities in ANC visits across different regions [49]. Education plays a crucial role by enhancing the capacity to comprehend and relate to health messages and raising awareness of the significance of maternal healthcare services. This improved understanding and awareness can lead to increased utilization and better health outcomes in maternal care.

Urban-rural inequality could also be minimized if rural women had the same autonomy as urban women in Bangladesh and Pakistan. Our descriptive analysis corroborates this, showing that urban women possess higher decision-making autonomy than their rural counterparts. The positive relationship between women’s autonomy and utilization of ANC is well documented, and women who have autonomy are more likely to utilize ANC services irrespective of region [50].

The factors significantly contributing to the inequality of quality ANC usage between urban and rural areas were consistent in both countries, but the extent of their contributions differed. After the wealth status in Pakistan, redistribution of women’s education could contribute more to eliminating inequality. With one of the lowest female literacy rates in Asia [51], over half of Pakistani rural girls do not complete elementary education, and nearly three-quarters do not pursue secondary education [52]. Therefore, improving women’s education in rural areas could significantly lower the disparity in using quality ANC. Conversely, in Bangladesh, the contribution of the distribution of women’s secondary and higher education to reducing urban-rural inequality is less pronounced compared to Pakistan. This may be due to the presence of various educational programs in Bangladesh which specifically focused on girls’ education, such as free education for girls up to high school and the Female Secondary Stipend and Assistance Program (FSSAP), which have significantly improved educational access for women in rural areas [53–55]. However, in Bangladesh, redistribution of media exposure could contribute more to alleviating inequality in quality ANC after wealth. According to a study conducted in Bangladesh [56], exposure to the media can significantly increase the usage of ANC services in rural regions.

The urban-rural inequality in the usage of quality ANC is disproportionately high in both countries and requires targeted strategies for elimination, especially by focusing on the factors that contribute most. If any significant improvements in quality ANC rates are to be attained, effective policies must be adopted to help rural women escape poverty. Despite the implementation of policies such as cash transfers, voucher schemes, and the elimination of user fees in an effort to increase maternal healthcare utilization and reduce inequality in maternal health in Pakistan and Bangladesh [9], inequality persists. To improve the utilization of maternal healthcare services among the underprivileged in the country, district/union council officials might detect eligible families in need, raise and maintain village funds to supplement government efforts and administer financial support so that it reaches the disadvantaged. Although reforms in the education system were initiated several times in Pakistan, the outcome has been limited [52]. Therefore, public, private, and philanthropic institutions and change-makers should pool resources to alleviate urban-rural inequality in education, especially among women. In Bangladesh and Pakistan, door-to-door campaigns and media coverage of ANC among underprivileged communities may foster awareness of the significance of maternal health services. Finally, the maternal program implementation should be location specific. It could be necessary to utilize mobile clinics in addition to expanding the number and quality of rural health facilities to make free maternity healthcare available in these locations. In particular, increasing healthcare utilization, especially ANC visits, is projected to result from the expansion of the Community-based Health Planning and Services (CHPS compound) effort in rural communities [34]. Since the CHPS compound program is highly integrated into the community and its health education program may involve educating pregnant women to seek ANC, implementing this in Bangladesh and Pakistan can significantly enhance ANC.

### Strengths and limitations

A key strength of our study is that it is the first to quantify and compare the drivers and magnitude of quality ANC inequalities in urban-rural settings between Bangladesh and Pakistan, both of which face similar challenges, including disparities in healthcare access, socio-economic inequalities, and limited healthcare infrastructure. Furthermore, we used nationally representative surveys of the same period and applied different decomposition techniques to check the robustness of the study outcome and finally employed a detailed decomposition analysis to identify the contribution of each factor to urban-rural inequality. These findings are crucial for Bangladesh, Pakistan and similar LMICs, including those aiming to reduce urban-rural inequality in MHS and achieve SDGs by 2030. Even with the strengths of this study, there are a few limitations to be aware of. The cross-sectional nature of our study precludes causal inferences regarding the factors contributing to the inequality. Therefore, caution is advised while interpreting the results. We acknowledge that the data on the utilization of quality ANC relies on self-reporting, which may introduce bias. Recall bias may also arise from including women who had birth three years before the survey. The inequality in using quality ANC services may be overestimated or underestimated due to this bias. To lessen this bias, an analysis was conducted on the most recent births three years before the survey. Additional research, based on data from other household surveys, is recommended to corroborate and expand upon our study’s findings.

## Conclusion

The study highlights a pronounced urban-rural inequality in the utilization of quality ANC services in Bangladesh and Pakistan, with a slightly greater inequality observed in Pakistan. Most of the overall difference in utilization of quality ANC is due to differences in characteristics between urban and rural women in these countries. Among the common significant predictors for both countries, wealth difference has the largest percentage of contribution, particularly differences in the rural-urban composition of women in the highest wealth quintile. In Pakistan, women’s education is the second largest contributor to reducing the urban-rural disparity in quality ANC, while in Bangladesh, both media exposure and husband’s education played notable roles. Future interventions to encourage the utilization of quality ANC in rural women of these countries should, therefore, emphasize increasing rural women’s household income. Additionally, allocating resources towards education in rural areas and expanding media coverage of ANC among underserved communities may enhance awareness of the importance of MHS and potentially reduce these inequalities.

## Supplementary Information

Supplementary Material 1.

Supplementary Material 2.

## Data Availability

The datasets analyzed during the current study are available on request in the DHS Program website: https://dhsprogram.com/data/available-datasets.cfm.
