# Supplementary material for: Comparison of urban-rural inequality in quality antenatal care among women in Bangladesh and Pakistan: a multivariable decomposition analysis
Source: Reprod Health. 2026 Jan 20;23:45. doi: 10.1186/s12978-026-02266-4 (PMC12905892; doi:10.1186/s12978-026-02266-4)
Supplement: Supplementary file 1 — Supplementary Material 1. [file 12978_2026_2266_MOESM1_ESM.docx]

**Mathematical Formulation of Decomposition Analysis**

The Oaxaca decomposition theory states that differences in the mean of an outcome between two groups can be explained by differences in the level or distribution of determinants (explained component) of the outcome and differences in the impact of these determinants on the outcome (unexplained component) [1].

Assume a regression model that links $Y$, the outcome variable, to a set of covariates, $X$ with a vector of coefficients, $\beta.$

$Y^{i}= \beta^{i}X^{i}$ where $i=$ urban, rural

The urban-rural mean difference between $\bar{Y}^{urban}$ and $\bar{Y}^{rural}$ can be written in two ways:

1. Oaxaca decomposition

$$\bar{Y}^{urban}-\bar{Y}^{rural}=\left( \bar{X}^{urban}- \bar{X}^{rural} \right)\beta^{urban}+\left( \beta^{urban}- \beta^{rural} \right)\bar{X}^{rural}$$

= $\Delta X\beta^{urban}+ \Delta\beta\bar{X}^{rural}$

= $E+C$ … (1)

Where $\Delta X$ is the mean difference between independent variables $\left( \bar{X}^{urban}- \bar{X}^{rural} \right)$ and $\Delta\beta$ is the difference between coefficients $\left( \beta^{urban}- \beta^{rural} \right).$

1. Blinder decomposition

$$\bar{Y}^{urban}-\bar{Y}^{rural}=\left( \bar{X}^{rural}- \bar{X}^{urban} \right)\beta^{rural}+\left( \beta^{rural}- \beta^{urban} \right)\bar{X}^{urban}$$

= $\Delta X\beta^{rural}+ \Delta\beta\bar{X}^{urban}$

= $E+C$ … (2)

Equations (1) and (2) are equivalent and determine the decomposition of the difference between the outcomes of the groups (urban/ rural). The two terms on the right-hand side represent the two components of the difference between the outcomes. The first component, $E$ (the explained component), is the average differences between the $X's$ of the urban and rural women and the second component, $C$ (the unexplained component), is the average difference of the $\beta's$, each of which is multiplied by weights. Generally, the first component was weighted by coefficients, while covariates weighted the second component.

The Oaxaca decomposition [2] in equation (1) used the high group (urban women with a higher rate of quality ANC utilization in this study) as the reference group, and the Blinder decomposition in equation (2) does the opposite, using the low group as the reference group (rural women in this study). The Oaxaca decomposition asserts that the outcome of the high group is in accord with their characteristics, and the low group is due to discrimination against them. In contrast, the Blinder decomposition considers that the outcome of the low group complies with their characteristics, and the high group is due to societal favoritism [3].

Other methods suggest utilizing weighted averages of the two groups as weights. According to Reimers [4], the weighted mean should be calculated as 0.5 (equal weights for the two groups: urban/ rural), although Cotton [5] says it should be the proportions of the two groups in the sample. Because the outcome of the decomposition is dependent on the weighting method used [6], this study performed a separate decomposition for each approach: Oaxaca, Blinder, Reimers, and Cotton. Regressions for urban and rural women were conducted separately, and the estimated coefficients and covariates were utilized to generate decompositions. Consistent results across weights exhibit the robustness of the findings.

**Reference:**

1. O'Donnell, O., et al., *Analyzing health equity using household survey data: a guide to techniques and their implementation*. 2008, Washington, D.C: World Bank. xi-xi.

2. Oaxaca, R., *Male-Female Wage Differentials in Urban Labor Markets.* International economic review (Philadelphia), 1973. **14**(3): p. 693-709.

3. Dewau, R., et al., *Urban-rural disparities in institutional delivery among women in East Africa: A decomposition analysis.* PLoS One, 2021. **16**(7).

4. Reimers, C.W., *Labor Market Discrimination Against Hispanic and Black Men.* The review of economics and statistics, 1983. **65**(4): p. 570-579.

5. Cotton, J., *On the Decomposition of Wage Differentials.* The review of economics and statistics, 1988. **70**(2): p. 236-243.

6. Kaiser, B., *Detailed decompositions in nonlinear models.* Applied economics letters, 2015. **22**(1): p. 25-29.
