# Supplementary material for: Comparison of urban-rural inequality in quality antenatal care among women in Bangladesh and Pakistan: a multivariable decomposition analysis
Source: Reprod Health. 2026 Jan 20;23:45. doi: 10.1186/s12978-026-02266-4 (PMC12905892; doi:10.1186/s12978-026-02266-4)
Supplement: Supplementary file 2 — Supplementary Material 2. [file 12978_2026_2266_MOESM2_ESM.docx]

| **Supplementary Table 1: Variable’s name, categorization, leveling and coding** | | |
| --- | --- | --- |
| **Outcome Variables** | **Description** | **Groups/ Levels (Codes)** |
| Quality Antenatal care (ANC) | Quality ANC is defined as: a woman has four or more ANC visits, of which at least one is with a medically trained provider and receives the basic components of ANC (blood pressure measurements, urine and blood sample taken) at least once. | ‘0’ = No, ‘1’ = Yes |
| **Independent variable** | **Description** | **Groups/ Levels (Codes)** |
| Place of Residence | Place/ Area of Residence | ‘1’ = Urban, ‘0’ = Rural |
| Woman’s Age (Years) | Age of the women in years during last live birth | ‘1’ = 15–19, ‘2’ = 20–34, ‘3’ = 35-49 |
| Women’s Education | Women’s level of education | ‘0’ = No formal education, ‘1’ = Primary education not completed, ‘2’ = Primary education completed (Grade 5), ‘3’ = Junior school completed (Grade 8), ‘4’ = Secondary or higher |
| Employment Status | Women’s currently employment/working status | ‘0’ = Not currently employed, ‘1’ = Currently employed  Notes: Employment is categorized as currently working (having worked in the past 7 days, including women who did not work in the past 7 days but who are regularly employed and were absent from work for leave, illness, vacation, or any other such reason) and not currently working. |
| Household Head | Women are head of their household | ‘0’ = No, ‘1’ = Yes |
| Media exposure | Women who were exposure to either radio, television newspaper or all three media at least once a week are considered regularly exposed to that form of media. | ‘0’ = No, ‘1’ = yes |
| Women’s autonomy | Women were considered to ‘have autonomy’ if they made decisions alone or jointly with their husbands regarding their own health care, made major household purchases and were able to visit family or relatives. | ‘0’ = No, ‘1’ = yes |
| Household Size | Number of members in the household | ‘1’ = 1-5 members, ‘2’ = 6 or more members |
| Wealth Quintile | DHS calculated the wealth index (WI) using data from the DHS databases on household asset ownership, household characteristics, household source of drinking water, and household sanitary facilities [26]. The principal components analysis (PCA) technique is used to construct the WI and assigns a score to each household based on chosen household assets. We used the constructed WI in the DHS to divide the population into wealth quintiles. | ‘1’ = Poorest, ‘2’ = Poorer, ‘3’ = Middle, ‘4’ = Richer, ‘5’ = Richest |
| Distance to health facility | Distance to a health facility | ‘0’ = Not a big problem, ‘1’ = Big problem |
| Husband’s Education | Husband’s level of education | ‘0’ = No formal education, ‘1’ Primary education not completed, ‘2’ = Primary education completed (Grade 5), ‘3’ = Junior school completed (Grade 8), ‘4’ = Secondary or higher |
| Last Live Birth Order | Order of the last live birth of the child | ‘1’ = First, ‘2’ = Second, ‘3’ = Third, ‘4’ = Fourth or Higher |
| Wanted Last Child | The last live birth was wanted/desired | ‘0’ = No, ‘1’ = Yes |
| Pregnancy Termination History | There was any pregnancy termination incidence | ‘0’ = No, ‘1’ = yes |

**Supplementary Table 2: Variance inflation factors (VIFs) of the variables included in the decomposition model**

| **Variable** | **VIF** | |
| --- | --- | --- |
|  | **Bangladesh** | **Pakistan** |
| Woman's age (years) |  |  |
| 20-34 | 1.70 | 3.81 |
| 35-49 | 1.72 | 4.16 |
|  |  |  |
| Women's education |  |  |
| Primary education not completed | 3.74 | 1.10 |
| Primary education completed | 2.95 | 1.22 |
| Junior school completed | 6.48 | 1.36 |
| Secondary or higher | 6.23 | 2.22 |
|  |  |  |
| Currently employed women | 1.10 | 1.06 |
|  |  |  |
| Women as household head | 1.02 | 1.02 |
|  |  |  |
| Women have media exposure | 1.37 | 1.32 |
|  |  |  |
| Women have autonomy | 1.09 | 1.12 |
|  |  |  |
| 1-5 members in household | 1.10 | 1.15 |
|  |  |  |
| Wealth quintile |  |  |
| Poorer | 1.73 | 1.76 |
| Middle | 1.89 | 2.08 |
| Richer | 2.17 | 2.52 |
| Richest | 2.79 | 3.34 |
|  |  |  |
| Distance is a big problem | 1.06 | 1.17 |
|  |  |  |
| Husband's education |  |  |
| Primary education not completed | 2.15 | 1.16 |
| Primary education completed | 2.02 | 1.30 |
| Junior school completed | 2.90 | 1.54 |
| Secondary or higher | 3.64 | 2.21 |
|  |  |  |
| Last live birth order |  |  |
| Second | 1.54 | 1.59 |
| Third | 1.54 | 1.64 |
| Fourth or higher | 1.69 | 2.76 |
|  |  |  |
| Has Pregnancy termination history | 1.04 | 1.04 |
| Mean VIF | 2.28 | 1.80 |

**Supplementary Table 3: Detailed decomposition of quality ANC by place of residence for Bangladesh and Pakistan, DHS 2017-18**

| **Decomposition** | **Bangladesh** | | | **Pakistan** | | |
| --- | --- | --- | --- | --- | --- | --- |
|  | **Coefficient** | **[95% CI]** | **Percent** | **Coefficient** | **[95% CI]** | **Percent** |
| Total difference | 0.2012** | [0.1706, 0.2317] | 100 | 0.2524** | [0.2172, 0.2876] | 100 |
| Explained | 0.1677** | [0.1405, 0.1948] | 83.35 | 0.2314** | [0.1930, 0.2698] | 91.68 |
| Unexplained | 0.0335 | [-0.0046, 0.0715] | 16.65 | 0.0210 | [-0.0295, 0.0715] | 8.32 |
| **Explained component = difference in characteristics (E)** | | | | | | |
| Woman's age (years) |  |  | 0.56 |  |  | -0.23 |
| 15-19 | 0.0009 | [-0.0011, 0.0028] | 0.43 | 0.0002 | [-0.0008, 0.0013] | 0.08 |
| 20-34 | -0.0001 | [-0.0010, 0.0008] | -0.06 | -0.0002 | [-0.0020, 0.0016] | -0.09 |
| 35-49 | 0.0004 | [-0.0005, 0.0013] | 0.19 | -0.0006 | [-0.0021, 0.0010] | -0.22 |
| Women's education |  |  | 0.21 |  |  | 23.23 |
| No formal education | -0.0005* | [-0.0009, -0.0000] | -0.23 | 0.0184* | [0.0017, 0.0352] | 7.30 |
| Primary education not completed | 0.0003 | [-0.0002, 0.0007] | 0.13 | 0.0021 | [-0.0023, 0.0064] | 0.82 |
| Primary education completed | -0.0001 | [-0.0023, 0.0022] | -0.03 | 0.0002 | [-0.0004, 0.0008] | 0.09 |
| Junior school completed | -0.0073** | [-0.0114,  -0.0031] | -3.62 | 0.0009 | [-0.0027, 0.0045] | 0.34 |
| Secondary or higher | 0.0080* | [-0.0003, 0.0157] | 3.96 | 0.0370** | [0.0205, 0.0536] | 14.68 |
| Currently employed women | 0.0012 | [-0.0065, 0.0090] | 0.61 | 0.0015 | [-0.0027, 0.0057] | 0.59 |
| Women as household head | -0.0011 | [-0.0056, 0.0033] | -0.57 | -0.0010 | [-0.0044, 0.0024] | -0.39 |
| Women have media exposure | 0.0179* | [0.0024, 0.0334] | 8.91 | 0.0237** | [0.0054, 0.0420] | 9.38 |
| Women have autonomy | 0.0042* | [-0.0002, 0.0086] | 2.09 | 0.0063* | [0.0014, 0.0111] | 2.48 |
| Wealth quintile |  |  | 58.92 |  |  | 43.67 |
| Poorest | 0.0218** | [0.0086, 0.0350] | 10.84 | 0.0473** | [0.0126, 0.0820] | 18.74 |
| Poorer | 0.0122 | [-0.0031, 0.0276] | 6.09 | 0.0017 | [-0.0127, 0.0161] | 0.67 |
| Middle | 0.0040 | [-0.0030, 0.0111] | 2.00 | 0.0015 | [-0.0048, 0.0079] | 0.61 |
| Richer | 0.0026 | [-0.0017, 0.0069] | 1.30 | 0.0172** | [0.0071, 0.0273] | 6.82 |
| Richest | 0.0778** | [0.0577, 0.0979] | 38.69 | 0.0425** | [0.0206, 0.0644] | 16.83 |
| Distance is a big problem | 0.0035 | [-0.0044, 0.0114] | 1.74 | 0.0045 | [-0.0092, 0.0181] | 1.77 |
| Husband's education |  |  | 9.65 |  |  | 7.68 |
| No formal education | 0.0002 | [-0.0032, 0.0036] | 0.09 | -0.0008 | [-0.0119, 0.0104] | -0.30 |
| Primary education not completed | 0.0034* | [0.0006, 0.0061] | 1.68 | 0.0003 | [-0.0008, 0.0014] | 0.11 |
| Primary education completed | 0.0005 | [-0.0005, 0.0014] | 0.22 | 0.0030* | [0.0005, 0.0054] | 1.17 |
| Junior school completed | 0.0001 | [-0.0015, 0.0017] | 0.05 | -0.0005 | [-0.0017, 0.0006] | -0.22 |
| Secondary or higher | 0.0153** | [0.0072, 0.0235] | 7.61 | 0.0175** | [0.0061, 0.0288] | 6.92 |
| Last live birth order |  |  | 1.25 |  |  | 3.51 |
| First | 0.0013* | [0.0000, 0.0026] | 0.64 | 0.0037** | [0.0012, 0.0061] | 1.45 |
| Second | -0.0006 | [-0.0020, 0.0008] | -0.29 | 0.0011 | [-0.0005, 0.0027] | 0.43 |
| Third | -0.0011 | [-0.0022, 0.0001] | -0.53 | -0.0001* | [-0.0002,  -0.0000] | -0.03 |
| Fourth or higher | 0.0029* | [0.0001, 0.0057] | 1.43 | 0.0042* | [-0.0030, 0.0081] | 1.66 |
| **Unexplained component = difference in coefficients (C)** | | | | | | |
| Woman's age (years) | | | | | | |
| 15-19 | -0.0032 | [-0.0210, 0.0146] | -1.59 | 0.0003 | [-0.0093, 0.0099] | 0.11 |
| 20-34 | 0.0056 | [-0.0456, 0.0567] | 2.76 | -0.0003 | [-0.0843, 0.0837] | -0.13 |
| 35-49 | 0.0005 | [-0.0058, 0.0068] | 0.24 | -0.0008 | [-0.0230, 0.0213] | -0.33 |
| Women's education | | | | | | |
| No formal education | 0.0001 | [-0.0082, 0.0081] | -0.04 | 0.0047 | [-0.0559, 0.0653] | 1.86 |
| Primary education not completed | 0.0056 | [-0.0101, 0.0213] | 2.78 | 0.0014 | [-0.0152, 0.0180] | 0.56 |
| Primary education completed | 0.0002 | [-0.0120, 0.0116] | -0.11 | -0.0044 | [-0.0247, 0.0158] | -1.76 |
| Junior school completed | 0.0150 | [-0.0204, 0.0504] | 7.44 | -0.0101 | [-0.0396, 0.0194] | -3.99 |
| Secondary or higher | -0.0133 | [-0.0331, 0.0066] | -6.60 | 0.0174 | [-0.0311, 0.0660] | 6.90 |
| Currently employed women | -0.0121 | [-0.0448, 0.0207] | -6.00 | -0.0089 | [-0.0457, 0.0279] | -3.53 |
| Women as household head | -0.0041 | [-0.0206, 0.0124] | -2.02 | 0.0104 | [-0.0234, 0.0441] | 4.10 |
| Women have media exposure | 0.0081 | [-0.0367, 0.0529] | 4.04 | 0.0627 | [-0.1080, 0.2333] | 24.83 |
| Women have autonomy | 0.0407 | [-0.0032, 0.0847] | 20.25 | 0.0158 | [-0.0409, 0.0725] | 6.27 |
| Wealth quintile | | | | | | |
| Poorest | -0.0180 | [-0.0465, 0.0104] | -8.96 | -0.0183 | [-0.1271, 0.0905] | -7.26 |
| Poorer | -0.0009 | [-0.0252, 0.0233] | -0.45 | 0.0396 | [-0.0745, 0.1537] | 15.69 |
| Middle | -0.0075 | [-0.0253, 0.0103] | -3.72 | 0.0015 | [-0.0325, 0.0355] | 0.58 |
| Richer | -0.0014 | [-0.0144, 0.0116] | -0.68 | 0.0078 | [-0.0262, 0.0417] | 3.08 |
| Richest | 0.0135 | [0.0022, 0.0248] | 6.71 | -0.0144 | [-0.0483, 0.0195] | -5.70 |
| Distance is a big problem | 0.0031 | [-0.0309, 0.0370] | 1.53 | 0.0110 | [-0.0562, 0.0782] | 4.36 |
| Husband’s education | | | | | | |
| No formal education | 0.0019 | [-0.0122, 0.0161] | 0.96 | 0.0297 | [-0.0585, 0.1178] | 11.75 |
| Primary education not completed | -0.0063 | [-0.0234, 0.0109] | -3.12 | -0.0029 | [-0.0161, 0.0104] | -1.13 |
| Primary education completed | 0.0020 | [-0.0105, 0.0146] | 1.02 | -0.0194 | [-0.0738, 0.0350] | -7.69 |
| Junior school completed | -0.0041 | [-0.0242, 0.0161] | -2.01 | 0.0045 | [-0.0219, 0.0308] | 1.77 |
| Secondary or higher | 0.0038 | [-0.0147, 0.0222] | 1.87 | 0.0283 | [-0.0507, 0.1072] | 11.21 |
| Last live birth order | | | | | | |
| First | 0.0094 | [-0.0198, 0.0386] | 4.66 | 0.0162 | [-0.0325, 0.0649] | 6.42 |
| Second | -0.0153 | [-0.0367, 0.0062] | -7.59 | 0.0091 | [-0.0230, 0.0412] | 3.62 |
| Third | 0.0095 | [-0.0047, 0.0237] | 4.72 | -0.0182 | [-0.0687, 0.0323] | -7.22 |
| Fourth or higher | -0.0039 | [-0.0175, 0.0097] | -1.95 | -0.0035 | [-0.0439, 0.0370] | -1.38 |
| Constant | 0.0051 | [-0.1031, 0.1132] | 2.51 | -0.1380 | [-0.5847, 0.3087] | -54.69 |
| Detailed decomposition through Blinder-Oaxaca multivariable decomposition devised by Powers et al.  ** Significance at 1% level, * Significance at 5% level and CI = Confidence Interval | | | | | | |
